# Training dynamically balanced excitatory-inhibitory networks

**DOI:** 10.1371/journal.pone.0220547

**Published:** 2019-08-08

**Authors:** Alessandro Ingrosso, L. F. Abbott

**Affiliations:** Zuckerman Mind, Brain, Behavior Institute, Columbia University, New York, New York, United States of America; University College London, UNITED KINGDOM

## Abstract

The construction of biologically plausible models of neural circuits is crucial for understanding the computational properties of the nervous system. Constructing functional networks composed of separate excitatory and inhibitory neurons obeying Dale’s law presents a number of challenges. We show how a target-based approach, when combined with a fast online constrained optimization technique, is capable of building functional models of rate and spiking recurrent neural networks in which excitation and inhibition are balanced. Balanced networks can be trained to produce complicated temporal patterns and to solve input-output tasks while retaining biologically desirable features such as Dale’s law and response variability.

## Introduction

Cortical neurons typically require only a small fraction of their thousands of excitatory inputs to reach firing threshold. This suggests an overabundance of excitation that must be balanced by inhibition to keep neurons within their functional operating ranges. An interesting suggestion is that this balance does not require fine-tuning of synaptic strengths, what we will call parametric balance, but rather occurs dynamically [1–8].

Dynamically balanced neural network models were originally introduced to account for the high variability of neural activity. Various forms of excitatory-inhibitory balance have been proposed for recurrent network models [9]. Because our aim is to construct networks that operate autonomously, we need to be in a strong-coupling regime, which means that the balance we discuss is of the ‘tight’ variety as defined by Hennequin et al. [9]. We subdivide tight balance into two classes, parametric and dynamic, depending on whether or not fine tuning of parameters is involved in maintaining the tight balance. This is important within the context of our study because, although parametrically balanced networks can be constructed and function as models, it is unclear whether the required fine tuning could be accomplished in a biological network. For this reason, we place emphasis on ways of training networks that result in a dynamically balanced configuration.

Variants of balanced networks have been used to model response selectivity [10, 11] and associative memory [12], but a general approach to task learning in these models has not previously been developed. The challenge is that learning can push a network that is initially in a dynamic balance into the parametrically balanced regime. We present approaches for training networks while retaining dynamic balance.

In addition to the issues with balancing outlined above, training networks with sign-constrained weights presents some technical challenges. Batch approaches to learning can handle sign constraints quite efficiently, but batch training of recurrent networks often leads to instabilities during testing, even when the training error is small [13, 14]. The use of an online strategy is critical to quench spontaneous chaotic fluctuations during training and to assure stability of the trained dynamics. These requirements demand fast learning algorithms capable of adjusting weights as the network is running. In previous work [13, 15, 16], this was achieved by using a recursive least squares (RLS) algorithm that has the favorable feature of constraining network dynamics while permitting fluctuations during training that are critical for post-training stability. Unfortunately, when sign-constraints are imposed, standard online training procedures, including RLS, are no longer viable. Here, we developed a fast sign-constrained online method that proves effective at training both rate and spiking balanced network models.

## Results

### Dynamically and parametrically balanced networks

The networks we consider are composed of either spiking neurons interacting via synaptic currents or so-called rate units. A task is generally specified by a set of desired output signals $F_{k}^{\text{out}}(t)$, for *k* = 1, 2, …*K*_out_ that are read out through channels *z*_*k*_. These signals can either be autonomously generated by the network or arise in response to *K*_in_ external inputs $F_{k}^{\text{in}}(t)$ entering the network through input weight vectors $w_{k}^{\text{in}}$. The input weights are generally chosen randomly and not subject to learning, whereas the readout weights, which are not sign-constrained, are trained using RLS. Recurrent weights are modified by an algorithm we discuss below. In rate models, $z_{k}=w_{k}^{\text{out}}\cdot \phi (x)$, where *ϕ*(*x*) is the rate activity for a unit with total input *x*. The equations of the *N* units of the network, for *i* = 1, 2, …, *N*, are

$$\begin{array}{c} \tau \frac{dx_{i}}{dt}=-x_{i}+\sum_{j=1}^{N}J_{ij}\phi (x_{j})+I+\sum_{k=1}^{K_{\text{in}}}w_{ik}^{\text{in}}F_{k}^{\text{in}} \end{array}$$ (1)

where *I* ∈ {*I*_E_, *I*_I_} is a vector of constant and uniform external currents into the E and I populations, and $w_{:k}^{\text{in}}$ are the weight vectors for each of the *K*_in_ input channels. To verify that our approach is general, we employ a variety of activation functions, e.g. halftanh (*ϕ*(*x*) = *θ*(*x*) tanh (*x*)), sigmoid (*ϕ*(*x*) = 1/(1 + exp(−*x*))) or ReLU (*ϕ*(*x*) = *θ*(*x*)*x*), where *θ* is the Heaviside step function (*θ*(*x*) = 1 when *x* > 0 and 0 otherwise).

For the spiking networks, we use leaky integrate-and-fire (LIF) dynamics (although good performances can be achieved with other neuronal models) of the form

$$\begin{array}{ccc} \tau_{\text{m}}\frac{dV_{i}}{dt} & = & -V_{i}+\sum_{j=1}^{N}J_{ij}s_{j}+\sum_{k=1}^{K_{\text{in}}}w_{ik}^{\text{in}}F_{k}^{\text{in}}+I \end{array}$$ (2)

$$\begin{array}{ccc} \tau_{s}\frac{ds_{i}}{dt} & = & -s_{i}+\tau_{s}\sum_{t_{i}^{\text{f}}<t}\delta (t-t_{i}^{\text{f}}) \end{array}$$ (3)

where *τ*_m_ is the membrane time constant (*τ*_m_ = 20 ms in all simulations) and $t_{i}^{\text{f}}$ is a list of the times when neuron *i* fired. When *V*_*i*_(*t*) reaches the spiking threshold *V*_th_ (usually set to 1) a spike is emitted and the voltage *V*_*i*_ is reset to *V*_res_ and kept constant for a period of time equal to the refractory period *τ*_ref_. We typically take either *τ*_ref_ = 2 ms or no refractoriness (*τ*_ref_ = 0), and *τ*_s_ = 50 ms or *τ*_s_ = 100 ms. The readouts for spiking networks are given by $z_{k}=w_{k}^{\text{out}}\cdot s$.

For networks with distinct excitatory and inhibitory neurons, the connection matrix ***J*** in Eqs (1) and (2), with elements *J*_*ij*_, is divided into 4 blocks, ***J***_EE_, ***J***_EI_, ***J***_IE_ and ***J***_II_, where the subscripts denote the type of post- and pre-synaptic neurons, respectively. For *N*_E_ excitatory and *N*_I_ inhibitory neurons, the dimensions of these submatrices are *N*_E_ × *N*_E_, *N*_E_ × *N*_I_, *N*_I_ × *N*_E_ and *N*_I_ × *N*_I_, respectively. To encompass both Eqs 1 and 2, we introduce the symbol *r* to signify either *ϕ*(*x*) or *s* and define the vectors ***r***_E_ and ***r***_I_ for excitatory and inhibitory neurons. Finally, we write each connection submatrix as the sum of its mean over elements and fluctuations around this mean: $J_{\text{XY}}={\bar{J}}_{\text{XY}}/\sqrt{N_{\text{Y}}}+\delta J_{\text{XY}}$, where x and y = e or i and ${\bar{J}}_{\text{XY}}$ is a scalar. We are interested in properties of the middle two terms in Eqs (1) and (2) and, for later analysis, we average these over both units (denoted by a square bracket) and time (denoted by an angle bracket). Thus, we define

$$\begin{array}{c} {\tilde{h}}_{\text{E}}={\bar{J}}_{\text{EE}}m_{\text{E}}\sqrt{N_{\text{E}}}+{\bar{J}}_{\text{EI}}m_{\text{I}}\sqrt{N_{\text{I}}}+{\text{I}}_{\text{E}}\;\text{and}\;{\tilde{h}}_{\text{I}}={\bar{J}}_{\text{IE}}m_{\text{E}}\sqrt{N_{\text{E}}}+{\bar{J}}_{\text{II}}m_{\text{I}}\sqrt{N_{\text{I}}}+I_{\text{I}}\;, \end{array}$$ (4)

where *m*_X_ = [〈***r***_X_〉], and

$$\begin{array}{c} c_{\text{E}}=\left[\delta J_{\text{EE}}\left\langle r_{\text{E}}\right\rangle +\delta J_{\text{EI}}\left\langle r_{\text{I}}\right\rangle \right]\;\text{and}\;c_{\text{I}}=\left[\delta J_{\text{IE}}\left\langle r_{\text{E}}\right\rangle +\delta J_{\text{II}}\left\langle r_{\text{I}}\right\rangle \right]\;. \end{array}$$ (5)

The existence and type of balance exhibited by a network can be characterized by the sizes of $\tilde{h}$ and *c*. We focus on cases with equal numbers of E and I neurons, so we refer to both *N*_E_ and *N*_I_ as being of order *N*, where *N* = *N*_E_ + *N*_I_ is the total number of units. For a network to function properly, the sum of $\tilde{h}$ and *c* in both the excitatory and inhibitory cases should be of order 1 despite the presence of the factors $\sqrt{N_{\text{E}}}$ and $\sqrt{N_{\text{I}}}$ in the expressions for $\tilde{h}$. In a standard dynamically balanced model, with random connectivity, this is assured by making I_E_ of order $\sqrt{N_{\text{E}}}$ and *I*_I_ of order $\sqrt{N_{\text{I}}}$. If appropriate balance stability conditions are met, *m*_E_ and *m*_I_ will dynamically adjust themselves to make both $\tilde{h}$’s of order 1, not of order $\sqrt{N}$. The condition determining these average rates is

$$\begin{array}{c} J^{\text{eff}}(\begin{array}{c} m_{\text{E}}\sqrt{N_{\text{E}}}\\ m_{\text{I}}\sqrt{N_{\text{I}}} \end{array})+(\begin{array}{c} I_{\text{E}}\\ I_{\text{I}} \end{array})∼0\;,\;\text{where}\;J^{\text{eff}}=(\begin{array}{cc} {\bar{J}}_{\text{EE}} & {\bar{J}}_{\text{EI}}\\ {\bar{J}}_{\text{IE}} & {\bar{J}}_{\text{II}} \end{array})\;, \end{array}$$ (6)

and the symbol ∼ implies equality to within a discrepancy of order 1 between terms of order $\sqrt{N}$. Our study is designed to find a learning procedure that assures that a similar cancelation occurs when the connection matrix is modified to make the network perform a task. The challenge is that, when learning adjusts the connection strengths, parametric rather than dynamic balance can arise.

One form of parametric balance occurs when *I*_E_ = *I*_I_ = 0 (or of order 1). In this case, if the trained recurrent weights scale like $1/\sqrt{N}$ and the network requires appreciable firing rates to do the task, Eq 6 must be evaded, because this would imply small rates if satisfied. A learning rule can achieve this by setting the determinate of *J*^eff^ to 0. This keeps $\tilde{h}$ of order 1, despite the lack of a balancing external current. Parametric balance can also occur when *I*_E_ and *I*_I_ are of order $\sqrt{N}$. Again, this is signaled by the learning rule setting det*J*^eff^ = 0 but, in this case, $\tilde{h}$ remains of order $\sqrt{N}$, that is, it does not balance. Instead, the overly large term $\tilde{h}$ is canceled, in this case, by terms of similar magnitude in *c*. The fine tuning required for the learning procedure to make the appropriate adjustments is why we call this parametrically balanced.

In our experience, many learning schemes result in connection matrices that realize a parametric rather than dynamic balance [12]. This comes about even if the initial connectivity ***J*** has a *J*^eff^ with determinant of order 1. One common way for this to occur is if learning sets the excitatory and inhibitory mean weight values so they are proportional to each other. We now show that an online learning scheme, combined with the appropriate regularization, can construct dynamically balanced models that solve a variety of tasks.

### Full-FORCE in E/I networks

**Fig 1:**
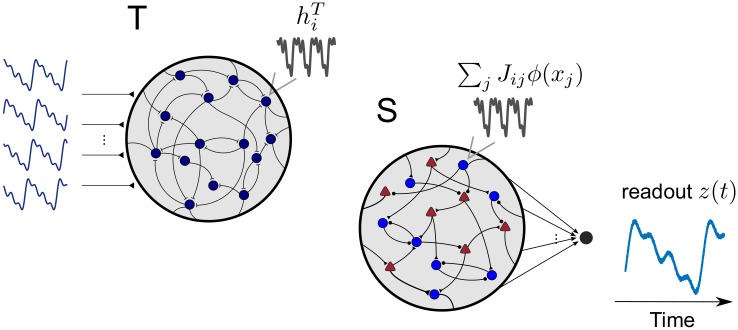
Schematic of the target-based method. Target currents $h_{i}^{\text{T}}\left(t\right)$ are produced by a balanced teacher network (T, Left) driven by the desired output. The student network (Right) is trained to reproduce the target currents autonomously. We train the recurrent weights of both the excitatory (E) and inhibitory (I) populations, together with the connections between them. A linear decoder ***w***^out^ is trained with a standard online method (RLS) to reproduce the prescribed output target from a readout of the neurons in the network.

We build upon a previously developed target-based approach for training rate and spiking networks [17–19] (Fig 1). A basic problem in network learning is that it is not clear what different units should do to help support the desired output. To solve this problem, we use a target-based approach [20]. If we knew the total synaptic input, ***Jr*** that each unit needed to function properly, finding the desired connection matrix ***J*** would be a simple least-squares problem. The trick is knowing what the targets are for these inputs. Full-FORCE is a scheme in which the target inputs are obtained from a second ‘teacher’ network [17–19].

In the full-FORCE scheme, the teacher network (T), which in the cases we consider is an E/I rate model, is driven by the desired output signals $F_{k}^{\text{out}}(t)$. This is done by adding a term $\sum_{k=1}^{K_{\text{out}}}w_{ik}^{T}F_{k}^{\text{out}}$ to Eq (1) with random weights $w_{ik}^{\text{T}}$ (we use superscript T to denote quantities associated with the teacher network). We then extract a set of target currents,

$$\begin{array}{c} h_{i}^{\text{T}}\;(t)=\sum_{j=1}^{N}J_{ij}^{\text{T}}\phi (x_{j}^{\text{T}}(t))+\sum_{k=1}^{K_{\text{out}}}w_{ik}^{\text{T}}F_{k}^{\text{out}}(t)\;, \end{array}$$ (7)

from the teacher network. The full recurrent synaptic matrix *J* of the network we are training (called the student network; variables without superscripts T are associated with the student network) is then trained to generate these target currents autonomously without any driving input. Specifically, for each neuron the training goal is to minimize the cost function, for a run of duration *t*_run_, *E* = ∑_*i*_ *E*_*i*_ with

$$\begin{array}{c} E_{i}=\frac{1}{t_{\text{run}}}\int_{0}^{t_{\text{run}}}dt{\left(h_{i}^{\text{T}}\;(t)-\sum_{j=1}^{N}J_{ij}\phi (x_{j}\left(t\right))\right)}^{2}+\alpha R_{i}\;. \end{array}$$ (8)

*R*_*i*_ is a regularization term to be discussed below. In our case, the expression in Eq (8) is minimized subject to sign constraints on the elements of the matrix *J*. The teacher networks we use are usually in a dynamically balanced configuration, but this is not essential.

In the original full-FORCE scheme [17, 18], the cost (8) is minimized using RLS but, as discussed above, this is not a viable procedure when sign constraints are imposed. Instead, we use bounded constrained coordinate descent (BCD) [21], which proves to be a fast and reliable strategy for training both rate and spiking models with sign constrained weights (Methods). The resulting learning algorithm is fast enough to effectively clamp the network dynamics close to the desired trajectory during training, suppressing chaos and assuring stability.

### Training dynamically balanced networks

**Fig 2:**
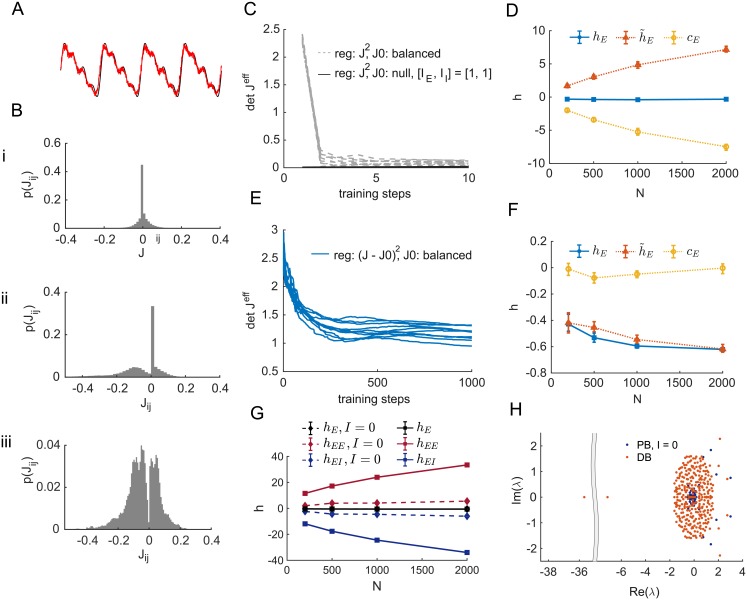
Trained balanced networks. A: Target output *F*^*out*^ (in black) for all the networks in this figure. Red curve is an example readout *z*(*t*) from a trained spiking network of *N* = 200 units. B: Histogram of recurrent weights in three prototypical trained rate networks (*N* = 300, *ϕ* = halftanh): i, Zero external current (*I*_E_ = *I*_I_ = 0) and L2 regularization; ii, $\left[I_{\text{E}},\;I_{\text{I}}\right]=\left(0.3\sqrt{N_{\text{E}}},0.4\sqrt{N_{\text{E}}}\right)$ and L2 regularization; iii, balanced initialization and J0 regularization, external currents as in ii. Regularization parameter *α* = 1.0 in all three cases. C: Time course of the determinant of the effective matrix *J*^eff^ during training of spiking networks of size *N* = 200 for *I* of order $\sqrt{N}$ (grey dashed lines) and *I* of order 1 (black line on horizontal axis). Both cases use L2 regularization. D: The full excitatory current and its two defined components (Eqs 4 & 5) as a function of N for a parametrically balanced network performing the task in panel A. E: Time course of the determinant of the effective matrix *J*^eff^ during training of spiking networks of size *N* = 200 for *I* of order $\sqrt{N}$ and J0 regularization. F: The full excitatory current and its two defined components (Eqs 4 & 5) as a function of N for a dynamically balanced network performing the task in panel A. Results in C-F are from ten different initializations of *J*^0^ or *J*^T^. G: The total average current onto E neurons (*h*_E_) and its excitatory (*h*_EE_) and inhibitory (*h*_EI_) components as a function of network size *N* for balanced networks (balanced initialization and J0 regularization, full lines) and networks trained with zero external currents (*I* = 0 and L2 regularization, dashed lines). H: Eigenvalue spectrum of the weight matrices *J* of two networks trained to perform the task in panel A (*N* = 200, *ϕ* = halftanh). Blue: Zero external current (*I*_E_ = *I*_I_ = 0) and L2 regularization; red: balanced initialization and J0 regularization.

For a given task, the distribution of synaptic weights after training depends on a variety of factors including the initial value of the *J* matrix, which we call *J*^0^, the choice of regularizer, and whether the network is tonically driven by large constant external current (*I* in Eqs (1) and (2)). We begin by considering a task in which the network must autonomously (meaning with time-independent input) generate the periodic output shown in Fig 2A. When no constant external current is present (*I* = 0), Eq (6) requires a parametric balance for any appreciable (larger than order $1/\sqrt{N}$) activity to exist in the network. The resulting parametrically balanced network can perform the task. We find that an extensive fraction of synaptic weights are set to zero by the training algorithm, so that the resulting networks display a connection probability ∼ 0.5 and a symmetric weight distribution (Fig 2Bi).

In the presence of constant external currents of order $\sqrt{N}$, the network has the potential to be dynamically balanced, but we find that, with a commonly used L2 weight regularization ($R_{i}=\sum_{j}\;J_{ij}^{2}$), the network also goes into a parametrically balanced configuration, though of a different form. This occurs regardless of the structure of the teacher network or the value of det *J*^eff^ for the initial weights *J*^0^. In this case, the weight distribution typically shows an extensive number of zero weights and a distribution of excitatory synapses that is approximately Gaussian but cut-off at zero (Fig 2Bii). The determinant of *J*^eff^ is small but, unlike the case with zero external current, it is not of order $1/\sqrt{N}$ (Fig 2C).

To determine the nature of the balance exhibited by the network trained with the L2 regularizer, we determined the scaling with *N* of various input terms, focusing on input to excitatory units. Both ${\tilde{h}}_{\text{E}}$ and *c*_E_ (Eqs 4 and 5) are of order $\sqrt{N}$, but they cancel to produce a total current $h_{\text{E}}={\tilde{h}}_{\text{E}}+c_{\text{E}}$ of order 1 (Fig 2D). This is indicative of parametric balance.

These results illustrate that dynamically balanced networks do not arise naturally from learning, even if the teacher network and the initial weight matrix of the student network are configured to be dynamically balanced and *I* is of order $\sqrt{N}$. The learning algorithm with L2 regularization tends to push the weight matrix to a parametrically balanced regime. We found a simple way to prevent this: choose *J*^0^ to satisfy the dynamically balanced condition (stable solution to Eq (6) with order 1 rates) and use regularization to keep *J* from straying too far from *J*^0^. The regularization that does this still uses an L2 norm, but on the difference between *J* and *J*^0^ rather than on the magnitude of *J*. Specifically, we define what we call the J0 regularizer by $R_{i}=\sum_{j}{\left(J_{ij}-J_{ij}^{0}\right)}^{2}$. With this regularizer, the weights after training display a Gaussian-like distribution (Fig 2Biii), block-wise average weights scaling as $1/\sqrt{N}$ and a *J*^eff^ determinant of order 1 (Fig 2E). Furthermore, the total current *h*_E_ and the two components we have introduced, ${\tilde{h}}_{\text{E}}$ and *c*_E_, are all of order 1 (Fig 2F). Thus, dynamically balanced networks trained by means of J0 regularization, even when they are fairly small, have average activities and currents in agreement with what is expected from a dynamically balanced regime.

To further examine the different forms of balance in these networks, we divide the total current $h_{\text{E}}={\tilde{h}}_{\text{E}}+c_{\text{E}}$ into a component arising from excitatory input (including *I*_E_), which we call *h*_EE_, and a component from inhibitory input, *h*_EI_. In the *I* = 0 parametrically balanced case, both these components and the total current are of order 1 (Fig 2G). In contrast, in the case of the dynamically balanced network generated using J0 regularization (Fig 2G), the total current is of order 1, while both its excitatory and inhibitory components scale like $\sqrt{N}$. Another difference between the parametrically balanced *I* = 0 (PB) and the dynamically balanced (DB) is seen in the spectrum of their connectivity matrices (Fig 2H): dynamically balanced networks show large negative eigenvalues [9].

**Fig 3:**
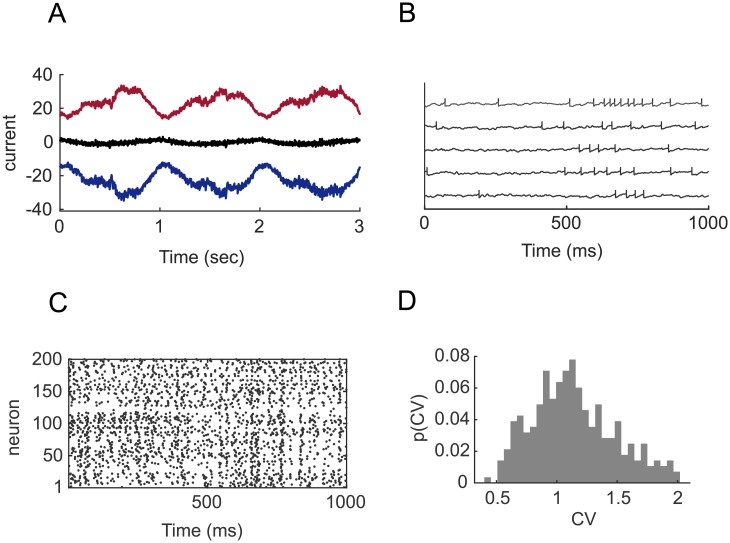
Dynamics in dynamically balanced trained spiking networks. A: Input currents onto a neuron in a spiking network trained to produce a superposition of 4 sine waves as in Fig 2A. Red curve: total excitatory current *h*_E_ + *I*_E_; Blue curve: inhibitory synaptic current *h*_I_; black curve: total current *h*. B: Voltage traces of 5 sample units the network with random fast synaptic currents (time constant 2 ms). C: Spike raster of 200 neurons for the network in B. D: Histogram of the coefficient of variation of interspike intervals across neurons for the network in B.

We can use BCD and J0 regularization to train dynamically balanced spiking networks as well (Fig 3). One common consequence of employing long synaptic time-scales is that a bursty spiking behavior emerges. The level of burstiness in trained networks can be varied by means of the *ω*_*h*_ parameter, that scales the intensity of the learned currents, generated by the slow synapses, with respect to the contribution provided by the random synapses with a fast time-constant (Methods). The irregularity of spiking in trained networks depends on the amplitude of the current fluctuations. To generate irregular spiking (Fig 3B–3D), we included random untrained fast-synapses (with synaptic time constant 2 ms; see [18]) and an average excess of inhibition. The level of spiking irregularity can be quantified by computing the distribution of coefficient of variations (CV) of interspike intervals across the neurons of the network (Fig 3D). The average CV≈1.

#### Perturbations in trained balanced networks

**Fig 4:**
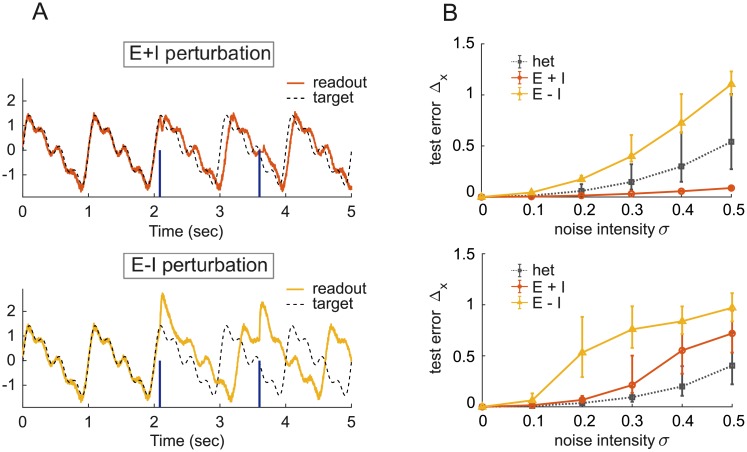
Response to perturbations in trained balanced networks. A: An E/I spiking network of size *N* = 200 trained on an oscillation task receives strong input pulses at random times (dark blue vertical lines), either in the E+I direction (top) or in the E-I direction (bottom). B: Median test error of two types of rate networks of size *N* = 200 trained to produce the same output signal as in A. Errorbars indicate 25% and 75% percentiles over 100 networks and 50 realizations of input white noise with intensity *σ*. The networks are driven either by *N* independent white noise inputs (black curve, legend: het) or by a single common white noise input in the E+I (red curve, legend E+I) or E-I (yellow curve, legend E-I) direction. Top: dynamically balanced network; bottom: parametrically balanced network with zero external input. Halftanh activation function, see Eq (9) for the definition of Δ_*x*_.

Balanced networks trained on autonomous oscillation tasks can suppress homogeneous perturbations in a way similar to the decorrelation effect mediated by the strong inhibitory feedback in such networks [3, 22]. As an example, we consider spiking networks trained to reproduce autonomously the periodic signal shown in Fig 2A. We constructed both dynamically and parametrically balanced examples of these networks and perturbed them at random times with 10 ms duration current pulses. These pulses come in two types, either identical for all neurons, or identical in magnitude but opposite in sign for excitatory and inhibitory neurons, with positive input to the excitatory neurons. We call these E+I and E-I perturbations, respectively. Balanced networks generally exhibit a strong resilience to E+I perturbations (Fig 4A, top) compared to external pulses in the E-I direction (Fig 4A, bottom). The latter produce a longer lasting transient and a subsequent larger phase shift in the network output. This response to temporary imbalance in the collective activity of the E and I populations is reminiscent of balance-amplified transients, previously described by a linear theory [23].

The role of inhibitory feedback is also apparent when a rate network is trained to produce the same rhythmic behavior. In this case, we perturbed the network with ongoing noise rather than with a transient. Homogeneous E+I input disturbances are cancelled by strong inhibitory recurrence in dynamically (Fig 4B, top) but not in parametrically (Fig 4B, bottom) balanced networks. E-I perturbations produce the strongest effect, and random heterogeneous perturbations produce similar effects in both networks, which are intermediate between E+I and E-I perturbations in the dynamically balanced case. E-I perturbations are somewhat amplified for the parametrically balanced case (Fig 4B, bottom). For these studies, we examined the effect not merely on the output, as in Fig 4A, but rather on the full network activity, defining

$$\begin{array}{c} \Delta_{x}=\frac{\int dt\sum_{i}{\left(x_{i}\left(t\right)-{\tilde{x}}_{i}\left(t\right)\right)}^{2}}{\int dt\sum_{i}{\left(x_{i}\left(t\right)\right)}^{2}}\;, \end{array}$$ (9)

where *x*(*t*) is the noiseless activity of the rate network and $\tilde{x}\left(t\right)$ the perturbed activity. We expect similar results to hold for spiking networks [5].

#### Autonomous activity in trained networks

**Fig 5:**
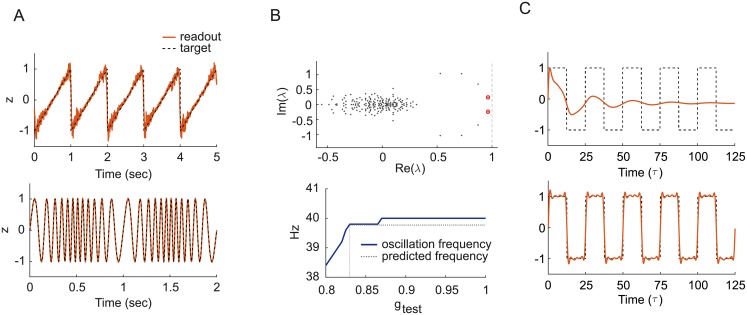
Nonlinear oscillations. A: Top: Balanced E/I spiking network of size *N* = 300 producing a sawtooth wave of frequency 1 Hz. Bottom: E/I rate network producing a frequency-modulated oscillation obtained by *F*^*out*^(*t*) = sin (*ω*(*t*)*t*) with *ω*(*t*) linearly increasing from 2*π* to 6*π* Hz for the first half of the oscillation period, then reflected in time around the midpoint of the period. Parameters: *N* = 500, *ϕ* = halftanh, trained using feedback (Methods, Δ*t*_*L*_ = 1 s). B: Top: Eigenvalue spectrum of *J*_test_*ϕ*′|_*x*0_ for a dynamically balanced rate network with sigmoid activation function trained to produce a square wave (*N* = 200, output frequency *f* = 0.04, *τ* = 1), for *g*_test_ = 0.8. The two red dots indicate the two conjugate eigenvalues λ_1,2_ with largest real value. Bottom: Oscillation frequency as a function of *g*_test_ comparing simulation results (solid curve) with approximate prediction (dashed lines). C: Readout signal with *g*_test_ = 0.8 (top) and *g*_test_ = 1.0 (bottom).

We found that the generation of oscillatory activity in trained network (such as that shown in Fig 5A) could be described by a simple mechanism, at least when a single frequency dominates that output pattern. After training, the spectrum of the synaptic matrix *J* usually shows a complex conjugate pair of eigenvalues with largest real part. This is not limited to target-based learning methods: we trained networks of different sizes with a variety of activation functions using back propagation through time (either employing stochastic gradient descent or ADAM [24]), and we consistently observed this phenomenon for different target readout signals of various frequencies. For differentiable activation functions, the oscillatory frequency is approximately predicted to be *f* = Im(λ_1_)/2*πτ*Re(λ_1_), where λ_1_ is one of the two complex eigenvalues with largest real part of the matrix *Jϕ*′|_*x*0_ (Fig 5B), with entries *J*_*ij*_ *ϕ*′(*x*_0,*j*_), and *ϕ*′|_*x*0_ is the derivative of the activation function computed at the (not necessarily zero) fixed point *x*_0_ from which the oscillations arise by means of a supercritical Hopf transition.

This analysis can be verified after training is completed by artificially lowering the effective gain of the obtained connectivity matrix *J* using a fictitious gain parameter *g*_test_ in the testing phase, such that *J*_test_ = *g*_test_*J*. Nonlinear oscillations arise at the critical value $g_{\text{test}}^{*}$ where the previously stable fixed point loses its stability as the two dominant conjugate eigenvalues cross the imaginary axis (Fig 5C). At the bifurcation, the frequency is controlled by the imaginary part of the dominant eigenvalues and the network dynamics is essentially two-dimensional. As *g*_test_ is increased, there is a small change of frequency of the readout signal as nonlinear effects start to grow and other frequencies and harmonics kick in (Fig 5B). This picture is consistent with previous work in random E/I separated rate models [25] as well as a recent study of low-rank perturbations to random connectivity matrices [26].

**Fig 6:**
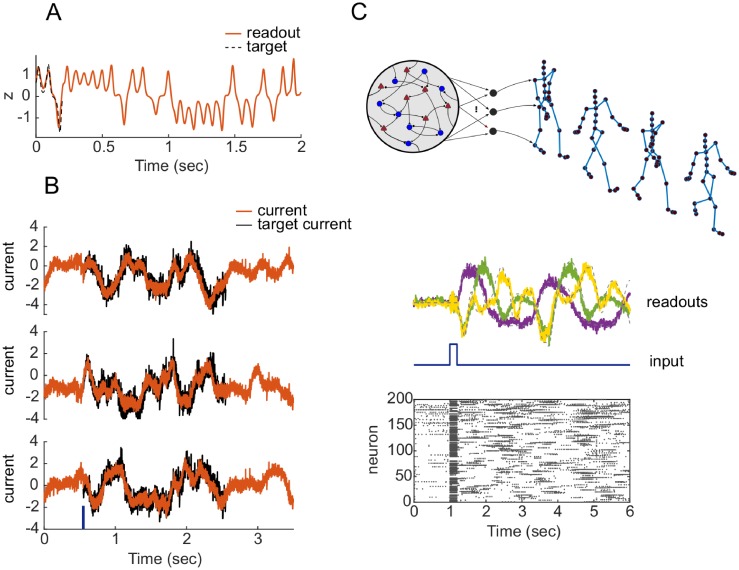
Learning chaotic trajectories and complex transient activity. A: Output of a rate network (*N* = 1000, halftanh activation function) trained to produce the time course of the first coordinate of a Lorenz attractor (*σ* = 10, *ρ* = 28, *β* = 2.67). B: Input currents onto three representative neurons in a balanced spiking network trained to reproduce innate current trajectories of duration 2 s after a brief stimulus (50 ms) at time 0.5 s. Network size *N* = 500, synaptic time constant *τ_s_* = 50 ms. C: Balanced E/I spiking network producing walking behavior in response to a strong input pulse of duration 100 ms. Top: a pictorial representation of the network with 56 distinct readouts (network size *N* = 300; synaptic time constant *τ_s_* = 50 ms). Middle: activity of three random readout units over the course of ∼ 6 s. Bottom: spike raster plot of 200 neurons in the network.

Balanced networks can also be trained to produce prescribed chaotic dynamics (like the Lorenz attractor in Fig 6A) or multiple complex quasi-periodic trajectories. In another task, inspired by the work of Laje, and Buonomano [15] in rate networks, and similar to recent extensions to the QIF spiking case in [27], we trained a spiking network to reproduce a desired transient dynamics in response to an external stimulus. To do so, we recorded innate current trajectories $h_{i}^{\text{T}}(t)$ generated by a randomly initialized LIF balanced network for a short period of time (2 sec) during its spontaneous activity. We then trained the same network to reproduce its innate current trajectories whenever a strong external input was applied (dark blue line in Fig 6B). The brief external pulse (50 ms) is able to elicit the target trajectory, after which the network naturally resumes its irregular activity. Finally, the example in Fig 6C shows an E/I spiking network instructed to generate the quasi-periodic dynamics of human walking behavior shortly after a 50 ms unitary pulse. We trained 56 linear decoders on the network activity to reproduce the time-course of each joint-angle from a human Motion-Capture dataset, as in [13, 28]. The average firing rate of the network is 20 Hz. A brief input pulse can trigger the motion generation from asynchronous spontaneous activity or reset the phase of a previously stable quasi-periodic dynamics.

#### Input-output tasks

**Fig 7:**
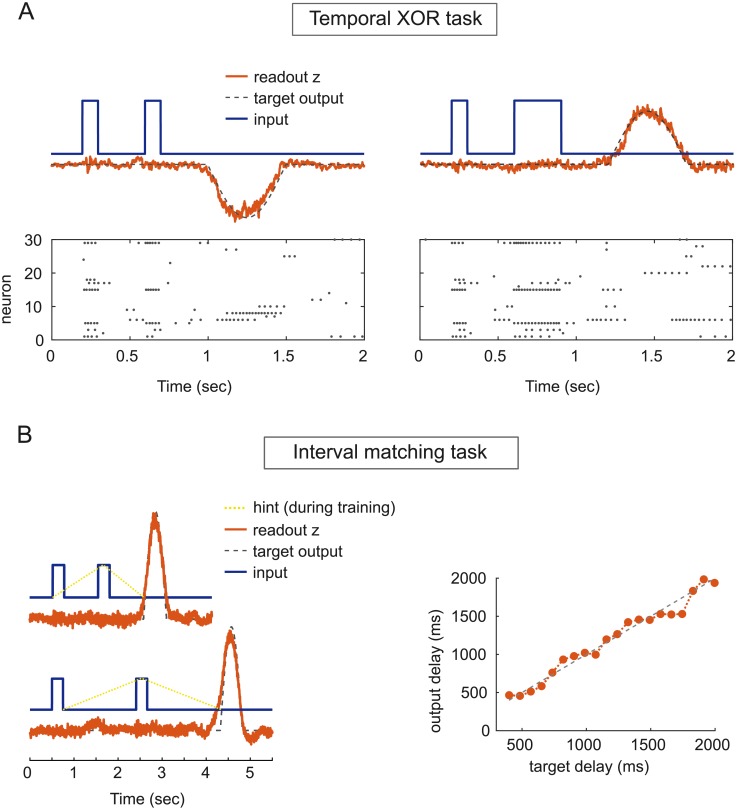
Input-output tasks. A: Example of output responses (red curves) of a balanced E/I spiking network trained on the temporal XOR task to two sets of input pulses (Blue curves) respectively coding for False-False (left) and False-True (right) Parameters: *N* = 1000, *τ_s_* = 50 ms. B: Interval matching task. Left: sample output (red curves) vs desired output (dashed black curves) from a spiking E/I network trained on the Interval Matching Task to two pairs of input pulses. Right: output delay vs target delay Δ*T* to randomly interleaved test input pulses.

Our learning procedure can also be employed to train dynamically balanced E/I networks capable of performing complex temporal categorization tasks. As our first example, a spiking network implements an exclusive OR function [18] anytime an appropriate sequence of inputs is presented, despite disturbance induced by its spontaneous asynchronous activity (Fig 7A). In each trial, the network is presented with two pulses of durations that are chosen randomly to be either short (100 ms) or long (300 ms), coding for the truth values 0 (False) or 1 (True). The network computes the XOR function of the two inputs and responds with an appropriate positive or negative readout signal (duration: 500 ms) after a delay period (300 ms). We used online BCD to train a balanced network of *N* = 1000 LIF neurons and measured the number of correct responses. The trained network responds promptly when the two impulses are presented at any random time over the course of its spontaneous activity and reaches a test accuracy of 96%.

As a second example, we construct an E/I spiking network to solve a more complex interval time-matching task, inspired by the “ready-set-go” task employed in [29]. This task has been solved previously using networks with unconstrained synaptic weights [19]. In this task, the network receives two brief input pulses separated by a random delay Δ*T*, and it is trained to generate a response after exactly the same delay, following the second pulse. As in the temporal XOR task described above, it is crucial here that the network retains information about the first pulse during the whole delay period in the absence of any external input. Especially for long delays Δ*T*, this task proves hard to solve. We therefore employ the heuristic technique of “hints” previously introduced in [19]: in each training epoch, the teacher network is provided with both a ramping up and decreasing input (dashed yellow line in Fig 7B, left) during the two relevant delay periods. An E/I network of *N* = 1000 spiking neurons produces accurate responses to random delays between 400 ms and 2 s (Fig 7B, right).

## Discussion

We have introduced a fast alternative to RLS that is capable of training sign-constrained rate-based and spiking network models and, in addition, has the promising features of good memory and computational requirements when dealing with E/I (and also sparse) models. We have shown that this fast target-based learning scheme can be used to train balanced networks of rate and spiking neurons for a wide variety of tasks. We described the conditions under which dynamically balanced networks can be obtained with the training procedure. We found that, in the absence of proper initialization and regularization, learning dynamics is attracted to regions of weight space with parametrically tuned connectivity, and we showed the impact of specific weight regularizations on the weight structure of trained networks, as well as their resilience to various external perturbations.

The regime in which we trained balanced networks to operate is an interesting one in which the computations relevant for a particular task are performed by dynamical modes orthogonal to the uniform modes that are constrained by the balance condition. We motivated our interest in training networks in the dynamically balanced regime by arguing that the order $1/\sqrt{N}$ fine tuning required for parametrically balanced networks might be hard for biological systems to maintain. We have looked for evidence of a higher sensitivity to weight perturbation in the parametrically balanced networks we constructed by a variety of methods. Unfortunately, trained recurrent networks of all types are sensitive to weight perturbations and, for the *N* values we used, we could not detect a strong difference in the robustness of these two network regimes. Thus, the motivation we introduced remains, at this point, speculative.

### Relation to other work

We have tackled the problem of training spiking neural networks to display prescribed stable dynamics or to solve cognitively relevant input-output tasks. A number of top-down approaches have been proposed to train functional models of spiking networks, e.g. the neural engineering framework [30], spike-coding [31] and nonlinear optimal control [28, 32]. These methods are elegantly formulated and effective. Interestingly, they solve a different task than what our procedure solves. These methods train the network to reproduce a prescribed dynamics, whereas our method trains a network to produce a particular trajectory generated by those dynamics. The resulting two networks look identical as long as the prescribed trajectory is being followed, but they generalize differently if the network deviates from this trajectory.

Some variations of RLS-based training have been introduced previously to construct functional models of E/I separated spiking networks. In [33], the authors employed a clipping procedure on top of a FORCE training method, which entails rank-1 updates to the original randomly connected recurrent network, while in [18] the authors used an off-line two step Full-FORCE procedure to train a large network performing an oscillation task. In a slightly different setting, the authors of [27] used Full-FORCE to train networks of quadratic integrate and fire neurons to reproduce prescribed synaptic drive, as well as spiking rate patterns in response to a brief strong stimulus. They provide an example of an E/I network with parametrically tuned effective connectivity and no external currents that tracks its own innate trajectories, recorded over the course of spontaneous activity. Sign constraints were imposed by eliminating updates of synapses that would pass out of the allowed ranges in a given epoch, and those synapses were then deleted in subsequent epochs. Backpropagation has been used successfully to train networks with separate excitatory and inhibitory units [34], and such networks have also been trained focusing on inhibitory plasticity [35].

### Conclusions

Credit-assignment is a major problem in training spiking networks, where differentiability issues limit the use of gradient-based optimization (but see [36–38]), which has proven very powerful in deep feed-forward architectures. Whereas in some approaches the credit assignment problem is tackled by relying on coding assumptions variably linked to optimality criteria, target-based approaches, both in the context of feed-forward [20] and recurrent models, provide a straightforward solution. As shown above as well as in a recent work [27], it is not essential for the teacher network to be a rate model, as long as it effectively acts as a dynamic reservoir that expands task dimensionality via its recurrency, therefore proving rich targets.

## Materials and methods

### Rate and spiking networks models

The weight matrix *J* is initialized by setting $J_{ij}=J_{\text{XY}}^{\text{eff}}/\sqrt{N_{\text{Y}}}+\Delta_{ij}$, where X and Y are the appropriate E and I labels corresponding to neurons *i* and *j*. Δ_*ij*_ is a random matrix with entries that are zero-mean Gaussian distributed with each column *j* having variance *g*^2^/*N*_Y_ (if any synapses violate constraints they get clipped at the first training iteration, otherwise we do not enforce any sparseness.). To construct a balanced teacher network, we use a non-negative activation function and appropriately choose block averages and external constant currents $i\propto \sqrt{N}$ for which the balance equation yields a solution with appreciable positive rates. In those cases where we seek to train spiking networks displaying irregular spontaneous activity with low rates, we further adjusted the random part Δ_*ij*_ so that ∑_*j*_ Δ_*ij*_ = 0 for each row *i*. By reducing quenched fluctuations in time-averaged activities for each neuron, this method ensures that spiking neurons trained on the teacher currents do not have abnormally low or large average activity.

Integration of ODEs is performed by the forward Euler method using an integration time-step not larger than Δ*t* = *τ*/20 for rate models and Δ*t* = 0.5 ms for spiking networks. We further scale down the integration time-step in all those case where large $J_{\text{XY}}^{\text{eff}}$ and strong external currents are employed.

### Learning algorithm

#### Bounded coordinate descent

When training a rate or a spiking network, we seek to match the incoming currents in the driven teacher $h_{i}^{\text{T}}(t)=\sum_{j}\;J_{ij}^{\text{T}}\phi (x_{j}^{\text{T}}(t))+\sum_{k}\;w_{ik}^{\text{out}}F_{k}^{\text{out}}(t)+I_{i}$ with those in the student: *h*_*i*_(*t*) = ∑_*j*_ *J*_*ij*_ *ϕ*(*x*_*j*_(*t*)) + *I*_*i*_ (for a rate student) or *h*_*i*_(*t*) = ∑_*j*_ *J*_*ij*_ *s*_*j*_(*t*) + *I*_*i*_ (for a spiking student). In training spiking networks, performance is virtually unchanged if one were to choose to match the activity *x*^*T*^(*t*) in the teacher rate network with the synaptic currents *h*(*t*) = *Js*(*t*) + *I* in the spiking network. We sometimes allow for an additional scaling and/or offset of the currents provided by the teacher network, so that the actual target currents are defined as $\omega_{h}h_{i}^{T}(t)+b_{h}$.

The teacher and spiking network are initialized with *x*_*i*_(0) or *v*_*i*_(0) i.i.d normal random variables. For input-output tasks, the two networks are initialized randomly at the beginning of each trial. For periodic tasks, we use a single trial encompassing multiple periods of the target signal.

Each neuron is trained independently and in parallel every Δ*t*_*l*_ (usually 1 ms), after a transient *T*_*d*_ = 20 *τ* to wash out the initial condition (we found this washout to facilitate learning especially for periodic tasks). We optimize the loss-function with an online strategy by means of Bounded Coordinate Descent (BCD). In our case, the method consists in updating, in parallel for each postsynaptic neuron *i*, each synapse *J*_*ij*_ one at a time by computing the optimal solution to the one-dimensional optimization problem where all other synapses *J*_*ik*_ for *k* ≠ *j* are kept fixed:

$$\begin{array}{c} J_{ij}\to \frac{J_{ij}C_{ii}+\alpha tW_{ij}+D_{ij}}{C_{ii}+\alpha t} \end{array}$$ (10)

where *α* is the regularization parameter in Eq 8, and *C* is proportional to the sample-covariance matrix of the activities $C_{ij}(t)=\sum_{\tau =0}^{t}s_{i}(\tau )s_{j}(\tau )$, which gets updated at each time-step by *C*_*ij*_ → *C*_*ij*_ + *s_i_s*_*j*_ (these equations are for the spiking case; for rate models *s_i_* is replaced by *ϕ*(*x_i_*)). The residual matrix *D*_*ij*_ is defined as $D_{ij}(t)=\sum_{\tau =0}^{t}s_{j}(\tau )(h_{i}^{\text{T}}(\tau )-h_{i}(\tau ))$. After each update with change Δ*J*_*ij*_, the *i*th row *D*_*i*:_ of the residual matrix *D* gets updated according to

$$\begin{array}{c} D_{i:}\to D_{i:}-\Delta J_{ij}C_{j:} \end{array}$$ (11)

where *C*_*j*:_ stands for the *j*th row of *C*. Setting $W_{ij}=J_{ij}^{0}$, where *J*^0^ is the initial weight matrix, we implement the J0 regularizer. Alternatively, *W*_*ij*_ = 0 corresponds to a simple L2 weight regularization. The amount of regularization is controlled by the parameter *α* (see Eq (8)).

**Fig 8:**
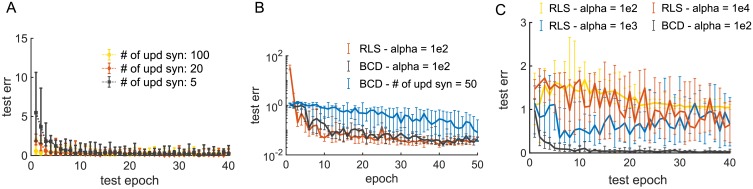
Some comparisons with RLS. A: Test error during training as a function of testing epoch for balanced networks of *N* = 200 LIF units trained on the oscillatory task in Fig 2A with BCD (*α* = 0.05). Each curve shows the results obtained when only a random subset of the incoming synapses onto each neuron gets updated. Networks were trained with feedback stabilization. Recurrent synaptic weights were updated every 20 time steps. The network was tested each 5 periods of the oscillations (1 sec). Each point is the median over 20 random initialization of both student and teacher networks. Bars represent 90% and 10% percentile. B: Test error as a function of training epoch for networks of *N* = 1000 Quadratic Integrate and Fire (QIF) neurons (for details see [27]) trained to reproduce their innate currents (task in Fig 6B) using Recursive Least Square or BCD. For both algorithms, recurrent synaptic weights were trained once each 25 time-steps (50 ms). Output weights were trained at each time-step via RLS. No feedback stabilization was employed in conjunction with BCD. For comparison with RLS, we employed a large value of *α* in BCD and did not normalize the first term of Eq (8) by *t*_run_, both in panel B and C. Parameters for QIF neurons: *τ_m_* = 10 ms, *τ_s_* = 100 ms, (similar to [27]), *τ*_ref_ = 2 ms, *dt* = 2 ms. C: Test error during training as a function of testing epoch for balanced networks of *N* = 200 LIF units trained on the oscillatory task in panel A, employing RLS with different values of the regularization parameter *α*. Results for BCD are shown for reference. The network was tested each 5 periods of the oscillations (1 sec). Each point is the median over 20 random initialization of both student and teacher networks. Bars represent 75% and 25% percentile.

The updating schedule of weight indexes *j* ∈ {1, 2, …*N*} can be either fixed or random at every step. For easier tasks, updating a random subset of incoming synapses at each time-step is enough to obtain good training performance (Fig 8A and 8B) at the price of slower convergence. We do not update the weights when this would violate the imposed sign constraints.

One of the benefits of BCD, compared to local optimization approaches (e.g. stochastic gradient decent), is its ability to keep the neural trajectory close to the target during training and prevent the network from shutting-down.

We note that coordinate descent proves a versatile method even beyond the sign-constrained case. For example, in updating incoming synapses to neuron *i*, it is easy to account for specific network topologies of the *J* matrix by selecting a relevant subset of rows/columns of the (symmetric) matrix *C* in the update Eq (10). Recursive Least Square with skipped updates for synapses out of the feasible region (we call this strategy Clipped-RLS) has a performance comparable to BCD (Fig 8A), but this strategy is memory-demanding for large network sizes, especially when dealing with dense topologies (where in-degree is O(N)). Clipped-RLS entails using *N* independent covariance matrices *P_i_*, one for each unit in the trained network, thus amounting to storing *N* × (*pN*)^2^ floating-point numbers (FPs). For comparison, BCD requires 2*N*^2^. Although we did not carry out systematic comparisons between Clipped-RLS and BCD, we found, for simple oscillatory tasks in balanced networks, that Clipped-RLS works best with bigger values of the regularization parameter *α* (Fig 8C), which penalizes strong deviation from the initial condition *J*^0^, and thus acts similarly to the J0 regularizer.

#### Regularization

In addition to the regularizations discussed in the text, we also experimented with a regularization of the form

$$\begin{array}{c} \sum_{\text{X}\in \{E,I\},j\in \text{X}}{\left(J_{ij}-\frac{\sum_{k\in \text{X}}\;J_{ik}}{N_{\text{X}}}\right)}^{2}\;, \end{array}$$ (12)

which controls the variance of the outgoing synaptic weights in each sub-population. For simple tasks, this typically produces inhibitory dominated networks with a non-singular *J*^eff^.

#### Feedback stabilization

We experimented with a feedback mechanism that can yield significant speed-up during training via a drastic reduction of the frequency of weight update 1/Δ*t*_*l*_. We found this method to be particularly effective in training periodic tasks. Specifically, during training we drive the student network with a modified current ${\tilde{h}}_{i}=h_{i}+\kappa (t)(h_{i}^{\text{T}}-h_{i})$. We use *κ*(*t*) = |*h* − *h*^T^|/(|*h*| + |*h*^T^|), with |*h*| the Euclidian norm of the vector *h* (although good training performance can be achieved with different metrics). The choice of an adaptive-gain feedback procedure frees from hyper-parameter optimization of the time-course of *κ*(*t*), which is usually taken to be a decreasing function of time. It is also instrumental in providing a minimal supervisory signal, thus allowing the student network to progressively exploit its own fluctuations over the course of training to build stability around the target trajectory.

When the feedback mechanism is in place, the minimization of the cost function Eq (8) can be carried out by quadratic programming once every Δ*t*_*l*_, using the matrices *C* and ${\tilde{D}}_{ij}(t)=\sum_{\tau =0}^{t}s_{j}(\tau )h_{i}^{\text{T}}(\tau )$. In preliminary experiments with simple periodic tasks, we found the interior-point method (quadprog.m in MATLAB) to work well. Results shown in the main text were obtained with BCD, which tends to be faster.

#### Testing

Test error is computed over a testing period *T*_*test*_ as

$$\begin{array}{c} E_{\text{test}}=\frac{\sum_{k=1}^{K_{\text{out}}}\sum_{t=0}^{{\text{T}}_{\text{test}}}{\left(z_{k}(t)-F_{k}^{\text{out}}(t)\right)}^{2}}{\sum_{k=1}^{K_{\text{out}}}\sum_{t=0}^{{\text{T}}_{\text{test}}}{\left(F_{k}^{\text{out}}(t)\right)}^{2}}\;. \end{array}$$ (13)

For input-output tasks, we randomly initialize the network state at the beginning of a test trial. For periodic targets *F*^out^(*t*), testing is interleaved with training, so that the spiking (rate) network state ***s*** (***x***) is usually close to the target trajectory. In this case, a sufficiently low test error usually implies the presence of a stable limit cycle, and the periodic output is reproduced, up to a phase shift, starting from any initial condition.

For the XOR task, during testing we defined a correct response when the normalized dot product of the readout *z* and *F*^out^, with *t* in the window of non-zero target, satisfied

$$\begin{array}{c} \frac{\sum_{t}z(t)F^{\text{out}}(t)}{\sqrt{\sum_{t}z^{2}(t)}\sqrt{\sum_{t}{\left(F^{\text{out}}(t)\right)}^{2}}}>0.5\;. \end{array}$$ (14)
